# Early Post-Transplant Red Blood Cell Transfusion Is Associated With an Increased Risk of Transplant Failure: A Nationwide French Study

**DOI:** 10.3389/fimmu.2022.854850

**Published:** 2022-05-31

**Authors:** Emilie Gaiffe, Dewi Vernerey, Laurent Bardiaux, Franck Leroux, Aurelia Meurisse, Jamal Bamoulid, Cécile Courivaud, Philippe Saas, Pierre Tiberghien, Didier Ducloux

**Affiliations:** ^1^Besançon University Hospital, Fédération Hospitalo-Universitaire Integrated Center for REsearch in inflammatory diseASes (FHU INCREASE), Besançon, France; ^2^Univ. Bourgogne Franche-Comté, Institut National De La Santé et de la Recherche Médicale (INSERM), Etablissement Français du Sang Bourgogne Franche-Comté, Unité Mixte De Recherche 1098 (UMR1098), RIGHT Interactions Hôte-Greffon-Tumeur/Ingénierie Cellulaire et Génique, Besançon, France; ^3^Methodology and Quality of Life in Oncology Unit, Centre Hospitalier Universitaire (CHU) Besançon, Besançon, France; ^4^Établissement Français Du Sang Occitanie, Toulouse, France; ^5^Institut National de la santé et de la recherche médicale Centre d'Investigation Clinique (INSERM CIC)-1431, Centre Hospitalier Universitaire Besançon, Besançon, France; ^6^Service de Néphrologie, Centre Hospitalier Universitaire Besançon, Besançon, France; ^7^Etablissement Français du Sang, La Plaine St Denis, France

**Keywords:** kidney transplantation, early transfusion, red blood cell, transplant failure, graft loss

## Abstract

**Background:**

Red blood cell (RBC) transfusions are frequently required in the early period after kidney transplantation. However, the consequences of RBC transfusions on long-term outcomes are largely unrecognized.

**Methods:**

We conducted a nationwide French cohort study involving all 31 French kidney transplant centers. Patients having received a first kidney transplant between January 1, 2002 and December 31, 2008 were identified through the national registry of the French BioMedecine Agency (*Agence de BioMédecine*). Number and date of RBC transfusions were collected from the national database of the French transfusion public service. The primary endpoint was transplant failure defined as graft loss or death with a functional graft.

**Results:**

Among 12,559 patients included during the study period, 3,483 (28%) were transfused during the first 14 days post-transplant. Median follow-up was 7.6 (7.5-7.8) years. Multivariable analysis determined that post-transplant RBC transfusion was associated with an increased risk in transplant failure (HR 1.650, 95%CI [1.538;1.771] p<0.0001). Both sensitivity and propension score analyses confirmed the previous result.

**Conclusions:**

Early red blood cell transfusion after kidney transplantation is associated with increased transplant failure.

## Introduction

Red blood cell concentrates (RBC) transfusions are widely used in the early post-transplant period (1). The reason being that there is a high prevalence of anemia which is caused by factors such as end-stage renal disease-associated erythropoietin deficiency, blood loss during surgery, and hemodilution (1). Also, anemia is a risk factor for death and graft loss after kidney transplantation (2–4).

Nevertheless, the impact of post-transplant RBC transfusions on transplant outcomes is less clear. In some studies, post-transplant RBC transfusion has been associated with the development of anti-HLA antibodies and antibody-mediated rejection in pre-sensitized patients (5–9).

However, only limited data suggest that RBC transfusion, *per se*, has long-term effects on graft or patient survival (10). A recent study suggests an association between RBCT and death-censored graft survival, but some pitfalls, including sample size, association with a negative control outcome, and too large of a temporal distribution of RBCT, limit the relevance of its results (10). Another study did not find an association between transfusion and graft outcome (11).

To better answer this critical question, we investigated the association between RBCT and kidney graft outcome in a very large national cohort linking databases from two national mandatory registers involving all patients having undergone kidney transplantation over a seven-year period in France.

## Materials and Methods

### Study Design

We conducted a national longitudinal study to investigate the association between early post-transplant RBC transfusion and transplant outcome. All transplant recipients in France are prospectively included in a mandatory registry (CRISTAL) administered by the French BioMedecine Agency (*Agence de BioMédecine*). Detailed patient characteristics were collected through this registry. Transfusion information was extracted from the national database of the French transfusion public service (*Etablissement Français du Sang*) which collects and prepares 100% of blood products issued in France. This database provides access to both pre- and post-transplant transfusions information. The two databases were merged for analyses. The method for building a single database from the cross of the two initial databases is described in the **Supplementary Methods**. This study was approved by the Committee for Personal Protection Great East II in Besançon (CPP: 13/07, 2013/02/25), by the Advisory Committee on Information Processing for Research in the Field of Health (CCTIRS, 13.138, 2013/03/19), and by French National Computers and Freedom Commission (CNIL, DR-2013-542, 2013/11/25).

### Patients and Blood Products

Patients were eligible for inclusion if they had received a first kidney transplant in one of the 31 French transplant centers between January 1^t^, 2002 and December 31, 2008. All data were extracted from the CRISTAL database. All RBC transfusion episodes that occurred from time of transplantation to 14 days following transplantation were considered. Patients with a graft survival of less than 15 days were excluded from analysis.

All RBC concentrates were produced by the Etablissement Français du Sang and underwent pre-storage deleukocytation (<1x10^6^ leucocytes/RBC unit). A transfusion episode was defined as encompassing consecutive transfusions where the interval between RBC transfusions did not exceed 48 hours (most often 1 to 2 RBC). Patients may have received multiple RBC transfusions from multiple donors.

### Study Outcomes

The primary endpoint was the failure free survival defined as the time between transplantation and transplantation failure. Patients known to be alive without transplantation failure were censored at the date of their last follow-up. A transplantation failure event was defined as graft loss or death in transplantation, whichever occurred first. A graft loss event was defined by the patient’s return to dialysis or retransplantation. Death in transplantation is defined by patient’s death with functional graft. Secondary endpoints were graft and patient survival. Graft survival was defined as the time between transplantation and graft loss. The data of graft survival were censored at the time of death or the last time of follow up for patients alive without graft loss. Patient survival was defined as the time between transplantation and death in transplantation. The data of patient survival were censored at the time graft lost or the last time of follow up for patients without graft loss.

### Statistical Analysis

Median (interquartile range), mean values (standard error), and frequency (percentage) were provided for continuous and categorical variables, respectively. Medians, means, and proportions were compared using Student’s t test and chi-square test (or Fisher’s exact test, if appropriate), respectively. Transplantation failure free survival, graft survival, and patient survival were estimated using the Kaplan-Meier method and described using median or rate at specific time points with 95%CI. Follow-up duration was calculated using a reverse Kaplan-Meier estimation (12). Cox proportional hazard models were performed to estimate HR and 95%CI for factors associated with transplant failure, graft failure, and death in transplantation.

The association of 13 baseline parameters with transplant failure was first assessed using univariate Cox analyses, and then parameters with p values of less than 0.05 were entered into a final multivariable Cox regression model, after considering collinearity among variables with a correlation matrix. When used continuously in the Cox model, a potential nonlinear relationship between predictors and transplant failure was first investigated using the fractional polynomials method to determine the best transformation for continuous variables and validated by the restricted cubic splines method with graphical evaluation (13, 14). The assumption of proportionality was checked by plotting log minus- log survival curves and by cumulative martingale process plots. Accuracy of the final model was verified regarding two parameters: discrimination and calibration. The predictive value and the discrimination ability of the final model were assessed with the Harrell’s C-index (15). Random samples of the population were used to derive 95%CI bootstrap percentile for the C-statistic. Calibration was assessed by visual examination of calibration plot. Internal validation of the final model was performed with a bootstrap sample procedure (16). To assess potential bias arising from missing data for parameters involved in the multivariable final model on their significance (P-value) and estimates (b and its standard error), a multiple imputation procedure with a Markov chain Monte Carlo method was performed using SAS MI and MIANALYZE procedure. The final multivariable Cox regression model for transplant failure was applied to graft loss and death in transplantation endpoints.

Several sensitivity analyses were performed. A full multivariable model including all parameters with p values of less than 0.05 in univariate analysis was undertaken to explore the reliability and the robustness of the final multivariable model for transplant failure. A propensity score approach to deal with potential heterogeneity in baseline characteristics between patients with and without transfusion in post transplantation was applied to assess the robustness of the association of post transplantation transfusion with transplant failure evaluated in the primary Cox multivariable analysis.

To address the potential confounding effect of unbalanced factors two methods were used: 1) inverse probability treatment weighting (IPTW), and 2) propensity score matching (17). HR and their 95%CI were estimated with the IPTW cox model. The propensity score matching technique, based on the caliper method, was performed to select two samples with well-balanced characteristics at time of transplantation (17).

All analyses were performed using SAS version 9.4 (SAS Institute, Cary NC) and R software version 2.15.2 (R Development Core Team, Vienna, Austria; http://www.r-project.org). P values of less than 0.05 were considered statistically significant, and all tests were two-sided. Details on the interpretation of important statistical concepts are given in the **Supplementary Methods**.

## Results

### Population Description

**Figure 1:**
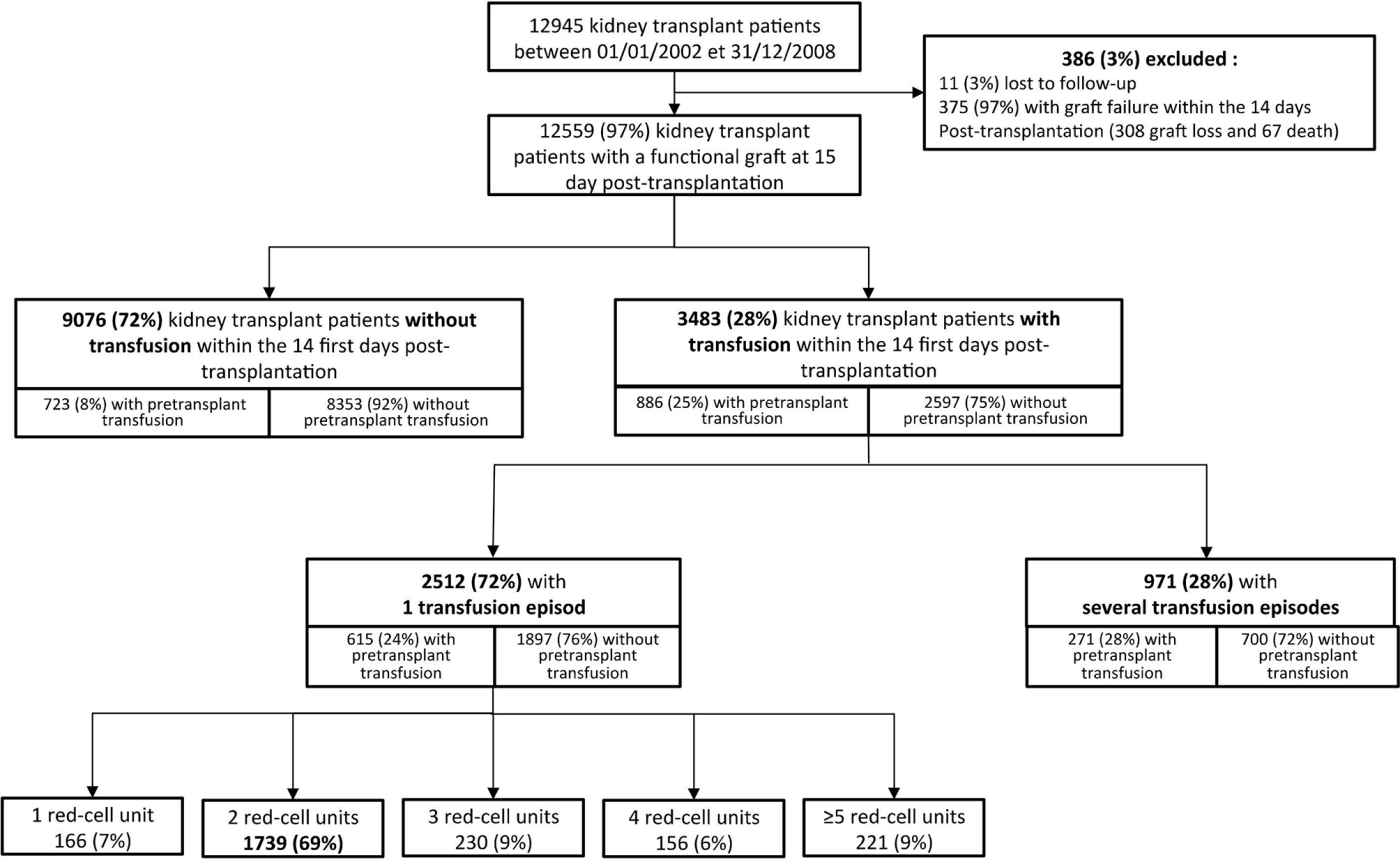
Study patient flow chart. A transfusion episode is defined as consecutive transfusions whose interval does not exceed 48 hours. Patients for whom follow-up does not reach 14 days post transplantation (post transplantation transfusion study period) are excluded from the study. The Figure also indicates whether patients received a transfusion in the pre and post transplantation settings.

The CRISTAL database identified 12,945 transplant patients having received a first kidney transplant between January 1, 2002 and December 31, 2008, among which 12,559 (97%) patients had a functional kidney allograft 15 days post-transplant (**Figure 1**). The linkage with the EFS database identified 3,483 (28%) patients having received an early post-transplant RBC transfusion. The majority of patients experienced one transfusion episode (n=2512, 72%), most often consisting of two RBC units (n=1739, 69%, **Figure 1**).

Transfused and non-transfused patients differed by several parameters (**Table 1A**). Nevertheless, patient follow-up was similar in both groups (7.8 95% confidence intervals, CI [7.6-8.0] *vs* 7.6 95%CI [7.4-7.7]; p=0.1523). Before transplantation, 1,609 patients had undergone transfusion (any history of transfusion), regardless of transfusion occurrence early after transplantation. Patients who received RBC after transplantation were more likely to have received transfusions before transplant (25.4% *vs* 8%; p<0.0001). **Table 1B** summarizes differences between patients having received RBC before transplantation and those who did not (**Table 1B**).

**Table 1:** Cohort characteristics description: (A) Patient characteristics according to the presence or absence of early post-transplantation transfusions, (B) Patient characteristics according to the presence or absence of prior transfusion.

A				
Characteristics	Overall patients (No. = 12559)	Patients without pre transplantation transfusion (No. = 10950)	Patients with pre transplantation transfusion in (No. = 1609)	p-value
**Recipient**				
Age^a^, year, mean (SD)	49.1 (13.3)	48.4 (13.2)	51.1 (13.3)	<0.0001
Median (IQR)	50.7 (39.7- 59.1)	49.8 (39.1- 58.2)	53.0 (41.5- 61.2)	<0.0001
Male gender^a^, No. (%)	7762 (61.8%)	5833 (64.3%)	1929 (55.4%)	<0.0001
BMI, kg/m², mean ± SD	24.2 (4.3)	24.2 (4.2)	24.1 (4.4)	0.1445
Median (IQR)	23.7 (21.0- 26.7)	23.7 (21.1- 26.8)	23.6 (20.9- 26.6)	0.0712
* Missing*	*2546*	*2009*	*537*	
BMI, kg/m²				
<18.5	638 (6.4%)	428 (6.0%)	210 (7.1%)	
18.5-24.9	5549 (55.4%)	3941 (55.8%)	1608 (54.6%)	
25.0-29.9	2829 (28.2%)	1986 (28.1%)	843 (28.6%)	
>30.0	997 (10.0%)	712 (10.1%)	285 (9.7%)	0.1867
* Missing*	*2546*	*2009*	*537*	
Dialysis antecedent, No. (%)	10744 (85.7%)	7747 (85.5%)	2997 (86.3%)	0.2753
* Missing*	*25*	*16*	*9*	
Hemodialysis, No. (%)				
No	1870 (17.8%)	1374 (18.1%)	496 (17.0%)	
Yes	8618 (82.2%)	6203 (81.9%)	2415 (83.0%)	0.1895
* Missing*	*256*	*170*	*86*	
**Causal Nephropathy** ^a^				
Glomerulopathy	3892 (31.0%)	2915 (32.1%)	977 (28.1%)	
Vascular nephropathy	903 (7.2%)	641 (7.1%)	262 (7.5%)	
Chronic interstitial nephropathy	587 (4.7%)	419 (4.6%)	168 (4.8%)	
Congenital	233 (1.8%)	175 (1.9%)	58 (1.7%)	
Polycystic	2137 (17.0%)	1677 (18.5%)	460 (13.2%)	
Uropathy	997 (7.9%)	744 (8.2%)	253 (7.3%)	
Diabetes	1225 (9.8%)	639 (7.0%)	586 (16.8%)	
Other	2585 (20.6%)	1866 (20.6%)	719 (20.6%)	<0.0001
**Transplantation**				
Anti-CMV antibodies, No. (%)				
+	4947 (60.7%)	3514 (59.5%)	1433 (63.8%)	
–	3205 (39.3%)	2393 (40.5%)	812 (36.2%)	0.0003
* Missing*	*4407*	*3169*	*1238*	
Overall HLA mismatch A/B/DR,				
mean ± SD	3.4 (1.3)	3.4 (1.3)	3.5 (1.3)	<0.0001
median (IQR)	4.0 (3.0-4.0)	4.0 (3.0-4.0)	4.0 (3.0-4.0)	<0.0001
* Missing*	*22*	*17*	*5*	
Anti-HLA immunization				
No	8434 (89.9%)	6242 (90.9%)	2192 (87.1%)	
Yes	948 (10.10%)	623 (9.1%)	325 (12.9%)	<0.0001
* Missing*	*3177*	2211	*966*	
Donor type^a^, No. (%)				
Living	926 (7.4%)	778 (8.6%)	148 (4.3%)	
Cerebrovascular death	11551 (92.0%)	8241 (90.8%)	3310 (95.0%)	
Other cause of death	82 (0.6%)	57 (0.6%)	25 (0.7%)	<0.0001
Cold ischemia time				
<12 h	1710 (17.9%)	1322 (19.0%)	388 (15.0%)	
12-24 h	5823 (61.1%)	4196 (60.4%)	1627 (62.9%)	
≥24 h	1999 (21.0%)	1427 (20.6%)	572 (22.1%)	<0.0001
* Missing*	*3027*	*2131*	*896*	
**Transfusion**				
Prior transfusion^a^, No. (%)	1609 (12.8%)	723 (8.0%)	886 (25.4%)	<0.0001
Recipient ABO blood group^*^, No. (%)				
A	5615 (44.7%)	4049 (44.6%)	1566 (45.0%)	
B	1173 (9.3%)	841 (9.3%)	332 (9.5%)	
O	5253 (41.8%)	3822 (42.1%)	1431 (41.1%)	
AB	518 (4.1%)	364 (4.0%)	154 (4.4%)	0.5886
Recipient D blood group, No. (%)	10276 (86.4%)	7392 (81.5%)	2884 (82.8%)	0.5854
* Missing*	*664*	*530*	*134*	
**Follow up**				
Transplantation follow-up (years, median (95%CI))	7.6 (7.5-7.8)	7.6 (7.4-7.7)	7.8 (7.6-8.0)	0.1523
**B**				
**Characteristics**	**Overall patients** **(No. = 12559)**	**Patient without pre transplantation transfusion (No. = 10950)**	**Patientwith pre transplantation transfusion in (No. = 1609)**	**P-value**
**Recipient**				
Age^a^, yr, mean (SD)	49.1 (13.3)	49.0 (13.2)	50.2 (13.8)	0.0012
Median (IQR)	50.7 (39.7- 59.1)	50.5 (39.6- 58.8)	52.3 (40.3- 60.7)	<0.0001
Male gender^a^, No. (%)	7762 (61.8%)	6819 (62.3%)	943 (58.6%)	0.0047
BMI, kg/m², mean ± SD	24.2 (4.3)	24.2 (4.3)	23.9 (4.5)	0.0325
Median (IQR)	23.7 (21.0- 26.7)	23.7 (21.1- 26.7)	23.5 (20.7- 26.7)	0.0157
* Missing*	*2546*	2208	338	
BMI, kg/m²,				
<18.5	638 (6.4%)	533 (6.1%)	105 (8.3%)	
18.5-24.9	5549 (55.4%)	4863 (55.6%)	686 (54.0%)	
25.0-29.9	2829 (28.2%)	2469 (28.3%)	360 (28.3%)	
>30.0	997 (10.0%)	877 (10.0%)	120 (9.4%)	0.0280
* Missing*	*2546*	2208	338	
Dialysis antecedent, No. (%)	10744 (85.7%)	9280 (84.9%)	1464 (91.2%)	<0.0001
* Missing*	*25*	*22*	*3*	
Hemodialysis, No. (%)				
No	1870 (17.8%)	1655 (18.3%)	215 (14.9%)	
Yes	8618 (82.2%)	7392 (81.7%)	1226 (85.1%)	0.0019
* Missing*	*256*	233	*23*	
**Causal Nephropathy** ^a^				
Glomerulopathy	3892 (31.0%)	3399 (31.0%)	493 (30.6%)	
Vascular nephropathy	903 (7.2%)	772 (7.1%)	131 (8.1%)	
Chronic interstitial nephropathy	587 (4.7%)	518 (4.7%)	69 (4.3%)	
Congenital	233 (1.8%)	208 (1.9%)	25 (1.6%)	
Polycystic	2137 (17.0%)	1897 (17.3%)	240 (14.9%)	
Uropathy	997 (7.9%)	906 (8.3%)	91 (5.7%)	
Diabetes	1225 (9.8%)	1029 (9.4%)	196 (12.2%)	
Other	2585 (20.6%)	2221 (20.3%)	364 (22.6%)	<0.0001
**Transplantation**				
Anti-CMV antibodies, No. (%)				
+	4947 (60.7%)	4317 (60.7%)	630 (60.6%)	
–	3205 (39.3%)	2795 (39.3%)	410 (39.4%)	0.9394
* Missing*	*4407*	3838	569	
Overall HLA mismatch A/B/DR				
mean ± SD	3.4 (1.3)	3.4 (1.3)	3.4 (1.3)	0.4055
median (IQR)	4.0 (3.0-4.0)	4.0 (3.0-4.0)	4.0 (3.0-4.0)	0.6048
* Missing*	*22*	17	5	
Anti-HLA Immunization				
No	8434 (89.9%)	7440 (90.8%)	994 (83.4%)	
Yes	948 (10.10%)	750 (9.2%)	198 (16.6%)	<0.0001
* Missing*	*3177*	*2760*	*417*	
Donor type^a^, No. (%)				
Living	926 (7.4%)	809 (7.4%)	117 (7.3%)	
Cerebrovascular death	11551 (92.0%)	10067 (91.9%)	1484 (92.2%)	
Other cause of death	82 (0.6%)	74 (0.7%)	8 (0.5%)	0.6961
Cold ischemia time				
<12 h	1710 (17.9%)	1495 (18.0%)	215 (17.8%)	
12-24 h	5823 (61.1%)	5070 (60.9%)	753 (62.3%)	
≥24 h	1999 (21.0%)	1759 (21.1%)	240 (19.9%)	0.5570
* Missing*	*3027*	2626	401	
**Transfusion**				
Post transplantation transfusion No. (%)^a^	3483 (27.7%)	2597 (23.7%)	886 (55.1%)	<0.0001
Follow up^a^				
Transplantation follow-up (years, median (95%CI))	7.6 (7.5-7.8)	7.8 (7.6-7.9)	7.0 (7.0-7.1)	<0.0001

a Missing, 0; Values of P<0.05 were considered statistically significant and all tests were two-sided.

BMI, Body Mass Index; CI, confidence interval; CMV, Cytomegalovirus; HLA, Human Leucocyte Antigen; IQR, Interquartile Range; SD, Standard Deviation; Tx, transplantation; yr, year.

The frequency of patients receiving at least one RBC unit after transplantation remained unchanged throughout the study period (**Supplementary Data **and **Table S1**).

### Early RBC Transfusion and Transplant Failure

In univariate analysis, age, male gender, dialysis before transplantation, primary renal disease, increasing number of HLA mismatch, CMV exposure, body mass index (BMI) higher than 24.9 kg/m^2^, donor type, and cold ischemia time > 12h were associated with transplant failure (**Table 2**). Both pre- and early post-transplant transfusions were also associated with transplant failure.

**Table 2:** Factors associated with transplant failure.

	Univariate analyses						Final multivariable Modeln = 7126 (2581 events)			
	N patients	N events		HR	95%CI	p value^b^	HR	95%CI	p value^b^	Internal validation
										HR 95% BCA Boot Strap Interval
Recipient age^a^	12559		3319	1.025	[1.022;1.027]	< 0.0001	1.023	[1.019; 1.027]	< 0.0001	[1.01857; 1.02743]
Recipient gender^a^										
Male	7762		2094	1			1			
Female	4797		1225	0.923	[0.860;0.991]	0.0262	0.899	[0.814; 0.992]	0.0342	[0.82285; 1.01397]
BMI, kg/m²,										
18.5-24.9	5549		1356	1			1			
<18.5	638		176	1.129	[0.965;1.321]		1.343	[1.115; 1.617]		[1.08972; 1.58730]
24.9.0-30.0	2829		750	1.136	[1.039;1.242]		0.975	[0.876; 1.086]		[0.87081; 1.08398]
>30.0	997		321	1.445	[1.280;1.633]	< 0.0001	1.245	[1.077; 1.439]	0.0003	[1.06544; 1.43427]
Missing	2546									
Dialysis antecedent										
No	1790		250	1			1			
Yes	10744		3047	2.082	[1.830;2.369]	< 0.0001	1.800	[1.527; 2.122]	< 0.0001	[1.52933; 2.13203]
Missing	25									
**Causal nephropathy** ^a^										
Glomerulopathy	3892		963	1			1			
Vascular nephropathy	903		332	1.729	[1.526;1.959]		1.363	[1.160; 1.602]		[1.14364; 1.56693]
Chronic interstitial	587		151	1.081	[0.911;1.284]		1.067	[0.860; 1.324]		[0.87372; 1.35873]
Nephropathy Congenital	233		51	0.858	[0.648;1.137]		0.949	[0.656; 1.373]		[0.61941; 1.33636]
Polycystic	2137		439	0.826	[0.737;0.924]		0.706	[0.605; 0.825]		[0.60277; 0.80856]
Uropathy	997		236	0.975	[0.846;1.124]		1.025	[0.848;1.238]		[0.79487; 1.20016]
Diabetes	1225		422	1.610	[1.436;1.806]		1.309	[1.125;1.524]		[1.11760; 1.50827]
Other	2585		725	1.204	[1.093;1.325]	< 0.0001	1.035	[0.907; 1.181]	< 0.0001	[0.90303; 1.17106]
**Transplantation**										
Anti-CMV antibodies										
–	3205		840	1						
+	4947		1430	1.159	[1.065;1.262]	0.0007				
Missing	4407									
Overall HLA. mismatch A/B/DR	12537		3314	1.078	[1.050;1.107]	< 0.0001	1.047	[1.009; 1.086]	0.0150	[1.00584; 1.09153]
Missing	22									
**Immunization**										
No	8434		2224	1			1			
Yes	948		314	1.311	[1.165;1.476]	< 0.0001	1.227	[1.060; 1.420]	0.0062	[1.061; 1.421]
Missing	3177									
Donor type^a^										
Living	926		131	1			1			
Cerebrovascular death	11551		3171	2.013	[1.690;2.397]		1.532	[1.192; 1.971]		[1.22496; 2.02935]
Other cause of death	82		17	2.146	[1.294;3.558]	< 0.0001	1.823	[1.051; 3.163]	0.0030	[0.96204; 3.31625]
Cold ischemia time min	9532		2578	1.000		< 0.0001				
Missing	3027									
Cold ischemia time										
<12 h	1710		299	1						
12-24 h	5823		1647	1.625	[1.437;1.838]					
≥24 h	1999		632	1.724	[1.502;1.978]	< 0.0001				
Missing	3027									
**Transfusion**										
Recipient blood group^a^										
A	5615		1503	1						
B	1173		300	0.978	[0.864;1.107]					
O	5253		1368	0.984	[0.915;1.059]					
AB	518		148	1.096	[0.926;1.298]	0.6419				
Recipient rhesus										
+	10276		2740	1						
–	1619		430	0.988	[0.893;1.094]	0.8226				
Missing	664									
Transfusion antecedent^a^										
No	10950		2765	1						
Yes	1609		554	1.635	[1.492;1.791]	< 0.0001				
Post transplantation transfusion^a^										
No	9076		2101	1						
Yes	3483		1218	1.650	[1.538;1.771]	< 0.0001				
Pre Post transplantation transfusion^a^										
No No	8353		1888	1			1			
Yes No	723		213	1.517	[1.317;1.748]		1.403	[1.261; 1.560]		[1.25971; 1.53334]
No Yes	2597		877	1.583	[1.462;1.716]		1.580	[1.306; 1.912]		[1.28960; 1.91767]
Yes Yes	886		341	2.146	[1.912;2.409]	< 0.0001	1.878	[1.613; 2.186]	< 0.0001	[1.62740; 2.15969]

The final multivariable Cox model was obtained by entering all parameters whose p value <0.05, excepting the parameters identified with a strong correlation (cold ischemia time is strongly correlated with donor type) and anti-CMV antibodies (not significant in the full multivariable model, **Supplementary Data, Table S3**, and lack of many data). For the multivariable model, pre- and post-transplant transfusions are analyzed together. The number of observations read is 9,976 and 2,581 events. Internal validation of the final model was performed with a bootstrap sample procedure.

a Missing, 0.

b Cox-proportional-hazard models used to estimate association of the parameters with transplantation success. Values of P<0.05 were considered statistically significant and all tests were two-sided.

BCA, Bias-corrected and accelerated; BMI, Body Mass Index; CI, confidence interval; CMV, Cytomegalovirus; HLA, Human Leucocyte Antigen; HR, Hazard Ratio.

A correlation matrix was used to detect significant correlations between investigated parameters and to select the variable the most clinically relevant and/or with less missing data (**Supplementary Data **and **Figure S1**). Cox analysis included ten independent risk factors: recipient age, gender, BMI, pre-transplant dialysis, primary renal disease, donor-recipient HLA mismatch(es), anti-HLA immunization, CMV exposure, donor type, cold ischemia time, and pre- and early post-transplantation transfusion (**Supplementary Data** and **Table S2**). Considering the very high rate of missing data regarding CMV exposure (n=4407/12949) and the lack of association with the outcome in the full multivariable model, this parameter was not included in the final multivariable model analysis (**Table 2**).

**Figure 2:**
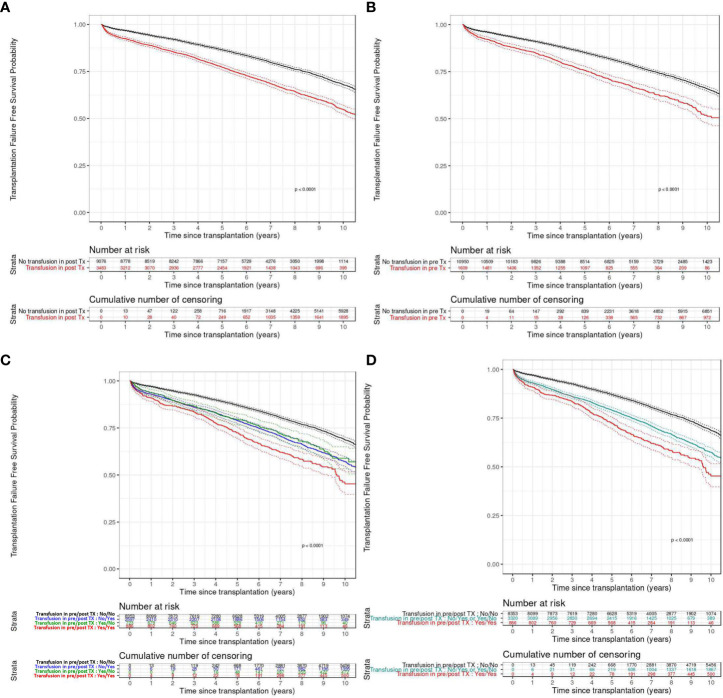
Transplantation failure free survival according to the pre-transplant **(A)**, post-transplant **(B)**, or both pre and post-transplant **(C, D)** transfusion status. **(A, B)** Patients transfused (in red) or not (in black). **(C)** Patients transfused before transplantation (in green), after transplantation (in blue), both (in red) or not (in black). **(D)** Patients transfused before and after transplantation (in red), before or after transplantation (in blue) transplantation or not (in black). TX, transplantation.

Final multivariable analysis revealed that pre- and post-transplant transfusions were associated with increased transplant failure (Hazard Ratio, HR 1.635, 95%CI [1.492;1.791] p<0.0001 and HR 1.650, 95%CI [1.538;1.771] p<0.0001, respectively) (**Table 2** and **Figure 2**).

### Sensitivity and Propensity Score Analyses

A multiple imputation analysis based on 500 imputed data sets provided similar results for all the variables (**Supplementary Data **and **Table S3**). The multivariable model exhibited good discrimination ability (Concordance Index, C-index= 0.619, 95%CI [0.610-0.628]). The calibration plots showed an optimal agreement between model prediction and actual observation for predicting transplant failure probability at one, three, five, and ten years (**Supplementary Data **and **Figure S2**). Moreover, transplantation failure free survival curves continue to diverge several years after transfusion (**Figure 2**). In the internal validation, uncertainties around hazard ratio measured with a bootstrapping procedure reflected the robustness of the final model (**Table 2**). The multivariable model that considers separately past history of transfusion and post-transplant transfusion showed that these two parameters were both independently associated with higher transplant failure (**Supplementary Data** and **Table S4**).

A propensity score analysis was performed (**Supplementary Data and Table S5A**). An inverse probability of treatment weighting (IPTW) analysis, post-transplant transfusion, remained associated with a higher risk of transplant failure (HR = 1.363; 95%CI [1.264-1.471]; p<0.0001) (**Supplementary Data ** and **Table S5B**). After propensity score matching of patients with similar characteristics (**Supplementary Data **and **Table S5C** and **Supplementary Data **and **Figure S3**), transplant failure rate was found to be higher in patients having received post-transplant RBC as compared to those not transfused after transplant (**Supplementary Data** and **Figure S4**).

### RBC Transfusion, Graft Loss, and Patient Death in Transplantation

**Figure 3:**
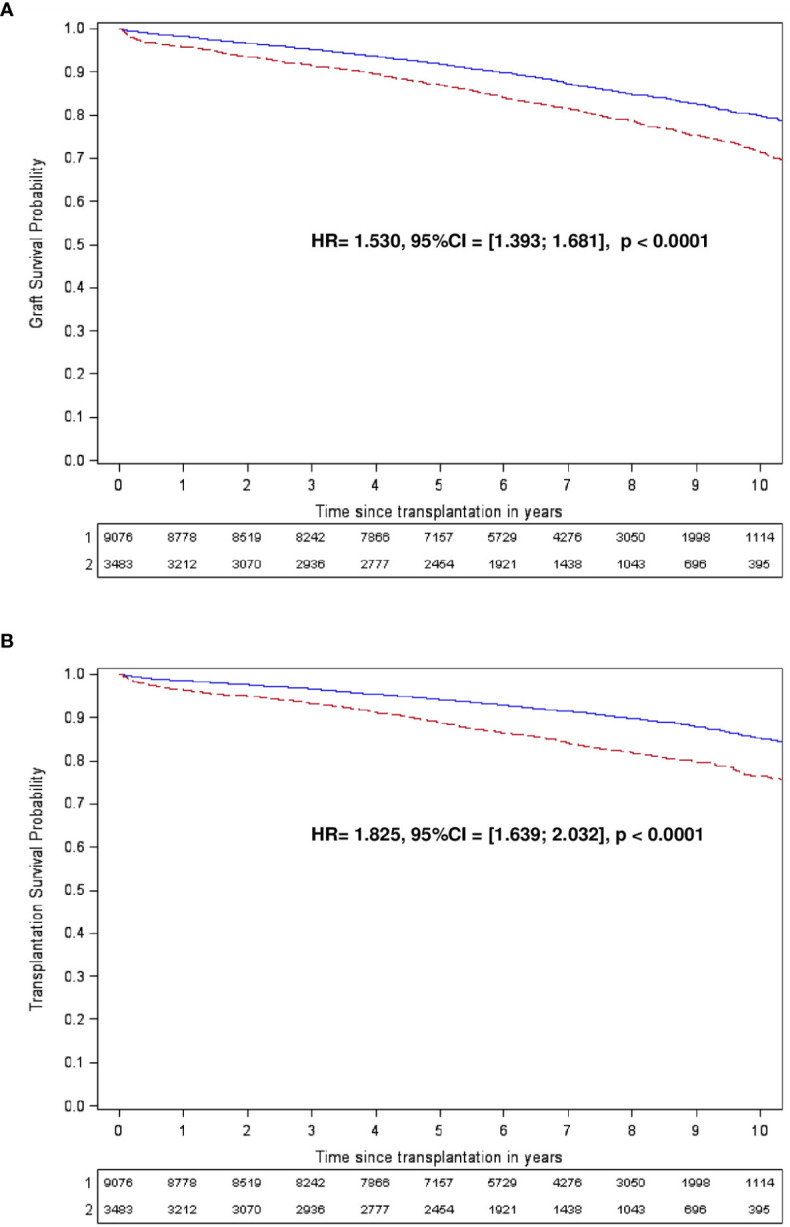
Kaplan-Meier graft **(A)** and transplantation **(B)** survival curves according to the post-transplantation transfusion. **(A, B)** Patients transfused (in red) or not (in blue) are followed for 10 years. TX, transplantation.

In multivariable analysis, both pre- and post-transplant transfusions were independently associated with an increased risk of graft loss and patient death in transplantation (**Table 3A, B**). Dialysis before transplantation, primary renal disease, and donor type were also associated with graft loss (**Table 3A**). Regarding death in transplantation, age, male gender, dialysis before transplantation and primary renal disease are significantly associated in a multivariable model (**Table 3B**). These results are also evidenced by Kaplan-Meier graft and patient survival curves according to the post-transplantation transfusion (**Figures 3A, B**).

**Table 3:** Multivariable analysis of parameters associated with graft loss (A) or death in transplantation (B).

A	Final multivariable model for graft loss		
	n = 7126 (1089 events)		
	HR	95% CI	p value**
Recipient age	0.997	[0.993; 1.002]	0.3125
Recipient gender			
Male Female	1 1.048	[0.923; 1.191]	0.4668
BMI, kg/m²,			
18.5-24.9 <18.5 24.9.0-30.0 >30.0	1 1.116 1.008 1.173	[0.883; 1.410] [0.872; 1.165] [0.959; 1.435]	0.3769
Dialysis antecedent			
No	1		
Yes	1.835	[1.478; 2.280]	< 0.0001
Causal Nephropathy			
Glomerulopathy Vascular nephropathy Chronic interstitial Nephropathy Congenital Polycystic Uropathy Diabetes Other	1 1.443 0.888 0.978 0.622 1.004 1.002 0.956	[1.168; 1.783] [0.657; 1.201] [0.645; 1.482] [0.502; 0.771] [0.800; 1.261] [0.812; 1.236] [0.805; 1.136]	< 0.0001
Overall HLA mismatch A/B/DR	1.046	[0.997; 1.099]	0.0676
Immunization			
No Yes	1 1.181	[0.974; 1.432]	0.0901
Donor type			
Living Cerebrovascular death Other cause of death	1 1.890 2.200	[1.366; 2.616] [1.091; 4.434]	0.0005
Pre Post Tx transfusion			
No No Yes No No Yes Yes Yes	1 1.740 1.389 1.794	[1.371; 2.207] [1.206; 1.600] [1.457; 2.209]	< 0.0001
**B**	**Final multivariable model for death intransplantation**		
	**n = 7126 (803 events)**		
	**HR**	**95% CI**	p value**
Recipient age	1.067	[1.059; 1.074]	< 0.0001
Recipient gender			
Male Female	1 0.732	[0.626; 0.857]	0.0001
BMI, kg/m²,			
18.5-24.9 <18.5 24.9.0-30.0 >30.0	1 1.713 0.957 1.367	[1.261; 2.326] [0.814; 1.124] [1.108; 1.688]	< 0.0001
Dialysis antecedent			
No	1		
Yes	1.700	[1.321; 2.190]	< 0.0001
Causal Nephropathy			
Glomerulopathy Vascular nephropathy Chronic interstitial Nephropathy Congenital Polycystic Uropathy Diabetes Other	1 1.272 1.346 0.719 0.902 0.992 1.888 1.173	[0.989; 1.638] [0.984; 1.841] [0.319; 1.623] [0.715; 1.137] [0.706; 1.393] [1.506; 2.367] [0.952; 1.445]	< 0.0001
Overall HLA mismatch A/B/DR	1.028	[0.973; 1.087]	0.3215
Immunization			
No Yes	1 1.308	[1.045; 1.637]	0.0189
Donor type			
Living Cerebrovascular death Other cause of death	1 1.159 1.596	[0.778; 1.725] [0.654; 3.898]	0.5611
Pre Post Tx transfusion			
No No Yes No No Yes Yes Yes	1 1.327 1.420 1.937	[0.966; 1.824] [1.208; 1.668] [1.550; 2.421]	< 0.0001

All parameters with p value <0.05 have been retained in models. (A) The number of observations read is 7126 and 1089 used. (B) The number of observations read is 7,126 and 803 used. **Cox-proportional-hazard models used to estimate association of the parameters with graft or patient survival. Values of P<0.05 were considered statistically significant and all tests were two-sided. BMI=Body Mass Index, CI, Confidence Interval; CMV, Cytomegalovirus; HLA, Human Leucocyte Antigen; HR, Hazard Ratio, Tx, Transplantation.

## Discussion

In a nationwide study including more than 12,000 patients with a long follow-up, RBC transfusion was associated with impaired graft and patient survival after kidney transplantation. Transfusions are frequently required in the early post-transplant. Indeed, just over a quarter of patients received RBC transfusion during the first 14 days following transplantation. This highlights that a number of patients are at risk of transfusion-induced complications.

We reported that early post-transplant RBC transfusion was associated with a worse prognosis after transplantation. Few prior studies reported on the effects of RBC transfusion after kidney transplantation. A single center study showed significant one-year graft survival reduction in kidney transplant recipients having received perioperative RBCT (8). Recently, a retrospective cohort study suggested that RBCT may increase the risk of death-censored graft loss (10). Nevertheless, because RBCT was considered as soon as on the first post-transplant day, graft loss due to surgical complications requiring RBCT were likely to drive the association. However, RBCT was also associated with a negative control outcome. Moreover, the authors analyzed RBCT occurring at any time of the transplant period, ignoring a differential effect with time of transplantation. Finally, the cohort was rather small. All of these aspects hamper the relevance of this study. As we excluded graft loss occurring within 14 days post-transplant, we probably excluded a large part of surgical-related graft loss having required RBCT.

The effect of transfusion, sustained with transplantation failure free survival curves, continues to diverge even years after the event. This suggests that transfusion may trigger a chronic process with long-term consequences. The mechanisms supporting the association between RBCT and graft loss are mainly based on transfusion-related DSA appearance. RBC units still contain white blood cells (WBC) even after deleukocytation. WBCs progressively disappear from RBC units in the first days of storage, but fresh RBC units containing greater amount of WBC expose patients to HLA immunization and subsequent humoral rejection. Ferrandiz et al. reported that the one-year incidence of donor-specific antibodies (DSA) was higher in patients who had undergone post-transplant transfusion (5). More recently, Hassan et al. suggested that transfusions elicit *de novo* HLA antibodies and decreases graft survival (14). Furthermore, the risk of antibody-mediated acute rejection increases in patients with-preformed DSA who received post-transplant transfusion (6). This hypothesis may explain the persistent long-term effects of early RBCT.

By contrast, several studies reported that transfusions do not induce DSA appearance or rejection (11, 18–23). Recently, Jalalonmuhali et al. reported that RBCT performed during the first week post-operative period were not associated with the development of *de novo* HLA-DSA, HLA-Ab or clinical rejection (21). Another study did not find any association between transfusion and antibody mediated acute rejection (22). Nevertheless, the number of patients included in this study was low, hampering robust conclusions about negative results. Another simultaneously published study did not find any effects of RBCT on graft outcomes as well acute rejection, graft loss, or death in transplantation (11).

The current study highly suggests that RBCT negatively affects transplant outcomes. These data have been obtained through the linkage of two national mandatory registers subject to regular in-house audits. The crossing method was precise and doubtful cases were all individually controlled. The results were tested in several sensitivity analyses and remained significant in all models. Unmeasured patients’ conditions (organ donor age, delayed graft function, and/or cause of RBCT) may be more associated with outcomes than transfusion by itself. Although we recognize that association does not imply causality, the robustness, the statistical independence, and the difference in magnitude of our findings should oblige us to reconsider the way we use this frequent and not so trivial treatment.

Our study reports that early post-transplant RBCT was associated with graft and patient survivals.

## Data Availability Statement

The raw data supporting the conclusions of this article will be made available by the authors, without undue reservation.

## Ethics Statement

The studies involving human participants were reviewed and approved by Committee for Personal Protection Great East II in Besançon (CPP: 13/07, 2013/02/25)by the Advisory Committee on Information Processing for Research in the Field of Health (CCTIRS, 13.138, 2013/03/19), and by French National Computers and Freedom Commission (CNIL, DR-2013-542, 2013/11/25). The ethics committee waived the requirement of written informed consent for participation.

## Author Contributions

EG and DD are the guarantors. EG, LB, PS, PT, and DD contributed to literature search, study design, setup of the study. EG, DV, LB, FL, AM, JB, CC, PS, PT, and DD participated to acquisition, analysis, or interpretation of data. EG, DV, AM, and DD realized statistical analysis. EG, DV, PT, and DD drafted the manuscript. EG, DV, LB, JB, CC, PS, PT, and DD revised the manuscript. The results presented in this paper have not been published previously in whole or part. This manuscript is an honest, accurate, and transparent account of the study being reported and no important aspects of the study have been omitted. The corresponding author attests that all listed authors meet authorship criteria and that no others meeting the criteria have been omitted. All authors contributed to the article and approved the submitted version.

## Funding

This study was supported by grants from Centre Hospitalier Universitaire de Besançon and from Etablissement Français du Sang (#2011-11 to PS).

## Conflict of Interest

LB, PS, and PT declare to be employed by the French transfusion public service (EFS).

The remaining authors declare that the research was conducted in the absence of any commercial or financial relationships that could be construed as a potential conflict of interest.

## Publisher’s Note

All claims expressed in this article are solely those of the authors and do not necessarily represent those of their affiliated organizations, or those of the publisher, the editors and the reviewers. Any product that may be evaluated in this article, or claim that may be made by its manufacturer, is not guaranteed or endorsed by the publisher.
