# Supplementary material for: Early Post-Transplant Red Blood Cell Transfusion Is Associated With an Increased Risk of Transplant Failure: A Nationwide French Study
Source: Front Immunol. 2022 May 31;13:854850. doi: 10.3389/fimmu.2022.854850 (PMC9197232; doi:10.3389/fimmu.2022.854850)
Supplement: Supplementary file 1 [file DataSheet_1.docx]

Red blood cell transfusion is correlated with an increase transplant failures: a French nationwide study

#

# **Supplementary Methods**

**Procedures**

All transplant patients are prospectively registered for the Cristal base. Both blood donor and transfused patients are registered for the Inlog registry. The crossing method aimed at identifying transplant patients from the Cristal database within the transfused patients from the Inlog database.

The Cristal database extract contained 11 items for identification: patient number, patient transplant waiting number, usual name, first name, surname, date of birth, gender, blood group, rhesus, date of transplant, and transplant team. Crossover items will not be retained for the study with the exception of transplant date, transplant team, blood group, rhesus, and gender.

ten recipient identification items were extracted from the EFS Inlog database: Inlog patient number, birth name, married name, first name, date of birth, the gender, blood type, rhesus, date of distribution, name of health facility, and name of service.

Patients were identified through a program created on the computer software SAS version 9.3. The crossing computer program analyzes and compares each variable used for crossing. The program considers whether data are identical or not (same letter and equal number of letters). The results are given in strata defining different degree of concordance:

- stratum A: association based on total concordance of all items,

- stratum B: association based on total concordance of available items but a name (for instance married name) is missing,

- stratum C, association is based on total concordance of items but names may be reversed (for instance birth name and married name),

- stratum D association is based on total concordance of items but name and first name are reversed.

Since concordance in strata A to D is based on identical fields, the results were not manually verified. In stratum E, a typographical error is present in one name but the lengths of the names are identical. Compared to stratum E, strata F, G, H, and I recapitulated the differences between strata B, C, and D and stratum A. In stratum X, only two variables are identical. Associations in stratum E to X were manually confirmed. All non-associated patients were gathered in stratum Z.

The results obtained during a test procedure (all the strata A to Z have been verified manually) showed that this program successfully crossed the two databases in 99.4% of cases including a manual verification of the crossings (stratum E to X) of 6.7%. The database was registered and declared to the National French Commission for bioinformatics data and patient liberty (CNIL).

**Statistical analysis interpretation**

The discrimination refers to the ability of separating patients with different prognosis. The C-index estimates the proportion of all pairwise patient combinations from the sample data whose survival time can be ordered according to whether the patient with the highest predicted survival is the one who actually survived longer (discrimination). The C-index (0 ≤ C ≤ 1) is a probability of concordance between predicted and observed survival, with C-index = 0.5 for random predictions and C-index = 1 for a perfectly discriminating model. In this study, the C-index calculation was repeated 1,000 times in random samples of the initial dataset with the use of bootstrap sampling procedures to derive 95% percentile confidence intervals for the C-index.

Calibration and goodness-of-fit refer to the ability to provide unbiased survival predictions in groups of similar patients. A prediction model is considered “well-calibrated” if the difference between predictions and observations in all groups of similar patients is close to 0 (perfect calibration).

Bootstrapping is the preferred simulation technique that was first described by Bradley Efron. 1 The original dataset is a random sample of patients being representative of a general population. Bootstrapping means generating a large number of datasets, each of which with the same sample size as the original one, by resampling with replacement (i.e., a previously selected patient may be selected again).

Internal validation is useful to obtain an honest estimation of the model performance for patients that are similar to those in the development sample and to indicate an upper limit to the expected performance in other settings. The bootstrap approach is the preferred technique to assess internal validity.

# **Supplementary References**

1 Efron B (1979) Bootstrap Methods: Another Look at the Jackknife. Ann Stat 7: 1-26

# **Supplementary Tables**

**Table S1 ǀ** Post-transplantation transfusion rate during the study period.

| Period | **Pourcentage of transfusion (n)** | **Total number of patients** |
| --- | --- | --- |
| 2002-2008 | 27.73 (3483) | 12559 |
| 2002 | 27.93 (429) | 1536 |
| 2003 | 30.58 (429) | 1403 |
| 2004 | 28.29 (488) | 1725 |
| 2005 | 26.95 (488) | 1811 |
| 2006 | 26.35 (501) | 1901 |
| 2007 | 27.30 (564) | 2066 |

Percentage of transplant patients transfused during the study period and each year.

**Table S2 ǀ** Multivariate analysis of parameters associated with transplant failure.

|  | **« Full » Multivariate Model**  **n = 5889 (1632 events)** | | |
| --- | --- | --- | --- |
|  | **HR** | **95% CI** | **p value**** |
| **Recipient age** | 1.023 | [1.018; 1.027] | < 0.0001 |
| **Recipient gender**  Male  Female | 1  0.896 | [0.805; 0.997] | 0.0434 |
| **BMI, kg/m²,**  18.5-24.9  <18.5  24.9.0-30.0  >30.0 | 1  1.311  0.932  1.243 | [1.073; 1.601]  [0.830; 1.047]  [1.062; 1.455] | 0.0005 |
| **Dialysis antecedent** |  |  |  |
| No | 1 |  |  |
| Yes | 1.654 | [1.389; 1.969] | < 0.0001 |
| **Causal Nephropathy** |  |  |  |
| Glomerulopathy  Vascular nephropathy  Chronic interstitial Nephropathy  Congenital  Polycystic  Uropathy  Diabetes  Other | 1  1.372  1.175  1.108  0.737  1.001  1.356  1.028 | [1.152; 1.634]  [0.935; 1.477]  [0.754; 1.626]  [0.624; 0.871]  [0.812; 1.233]  [1.151; 1.597]  [0.891; 1.187] | < 0.0001 |
| **Anti-CMV antibodies** |  |  |  |
| **-** | 1 |  |  |
| **+** | 1.065 | [0.961; 1.180] | 0.0181 |
| **Overall HLA mismatch A/B/DR** | 1.048 | [1.008; 1.090] | 0.0181 |
| **Immunization**  No  Yes | 1  1.242 | [1.065; 1.449] | 0.0058 |
| **Donor type**  Living  Cerebrovascular death  Other cause of death | 1  1.285  1.610 | [0.927; 1.779]  [0.843; 3.075] | 0.2277 |
| **Cold ischemia time**  <12 h  12-24 h  ≥24 h | 1  1.293  1.443 | [1.070; 1.563]  [1.173; 1.774] | 0.0022 |
| **Pre Post Tx transfusion**  No No  Yes No  No Yes  Yes Yes | 1  1.545  1.339  1.872 | [1.256; 1.901]  [1.195; 1.501]  [1.582; 2.213] | < 0.0001 |

All parameters with p value <0.05 have been retained in the model. The number of observations Read is 12559 and 5889 used. **Cox-proportional-hazard models used to estimate association of the parameters with transplantation success survival. Values of P<0.05 were considered statistically significant and all tests were two-sided. BMI=Body Mass Index, CI=Confidence Interval, CMV=CytoMegaloVirus, HLA=Human Leucocyte Antigen, HR=Hazard Ratio, Tx=Transplantation.

**Table S3 ǀ** Multiple imputation analysis for final multivariate model.

|  | **Complete-subject analysis**  **(N = 7126 / events = 1892)** | | | **Multiple imputation analysis**  **(N = 12559 / events = 3319)**  **(MCMC, N = 500 imputed dataset)** | | |
| --- | --- | --- | --- | --- | --- | --- |
|  | **ß** | **SE** | ***P*** | **ß** | **SE** | ***P*** |
| **Recipient age** | 0.02279 | 0.00199 | < 0.0001 | 0.021870 | 0.001512 | < 0.0001 |
| **Recipient gender**  Male  Female | -0.10658 | 0.05032 | 0.0342 | -0.110859 | 0.038056 | 0.0036 |
| **BMI, kg/m²,**  18.5-24.9  <18.5  24.9.0-30.0  >30.0 | 0.29490  -0.02521  0.21907 | 0.09488  0.05484  0.07397 | 0.0019  0.6457  0.0031 | 0.190081  -0.065409  0.109958 | 0.126402  0.107647  0.113902 | 0.1327  0.5435  0.3344 |
| **Dialysis antecedent**  No  Yes | 0.58778 | 0.08396 | < 0.0001 | 0.620394 | 0.066362 | < 0.0001 |
| **Causal nephropathy**  Glomerulopathy  Vascular nephropathy  Chronic interstitial nephropathy  Congenital  Polycystic  Uropathy  Diabetes  Other | 0.30972  0.06506  -0.05239  -0.34751  0.02471  0.26950  0.03429 | 0.08246  0.11010  0.18848  0.07936  0.09646  0.07757  0.06749 | 0.0002  0.5546  0.7810  < 0.0001  0.7978  0.0005  0.6114 | 0.347699  0.013362  0.045104  -0.268142  0.115362  0.360586  0.109377 | 0.064591  0.088221  0.144295  0.058588  0.073274  0.059535  0.049573 | < 0.0001  0.8796  0.7546  < 0.0001  0.1154  < 0.0001  0.0274 |
| **Overall HLA mismatch**  **A/B/DR** | 0.04546 | 0.01869 | 0.0150 | 0.052359 | 0.013910 | 0.0002 |
| **Immunization**  No  Yes | 0.20428 | 0.07457 | 0.0062 | 0.200651 | 0.061925 | 0.0012 |
| **Donor type**  Living  Cerebrovascular death  Other cause of death | 0.42691  0.60085 | 0.12840  0.28101 | 0.0009  0.0325 | 0.305416  0.432961 | 0.091176  0.259007 | 0.0008  0.0946 |
| **Pre Post transplant transfusion**  No No  Yes No  No Yes  Yes Yes | 0.45765  0.33832  0.63006 | 0.09711  0.05426  0.07750 | < 0.0001  < 0.0001  < 0.0001 | 0.392409  0.364328  0.609322 | 0.072655  0.041617  0.059782 | < 0.0001  < 0.0001  < 0.0001 |

Complete-subject analysis for the final model of factors associated with transplant failure (N= 7126) and multiple imputation analysis to assess potential bias arising from missing data for parameters involved in the multivariate final model with 500 imputed dataset. BMI=Body Mass Index, HLA=Human Leucocyte Antigen, MCMC=Markov chain Monte Carlo, SE=Standart Error.

**Table S4 ǀ** Multivariate analysis of parameters associated with transplant failure.

|  | | **Final multivariate Model**  **n 7126 = (2581events)** | | |
| --- | --- | --- | --- | --- |
|  |  | **HR** | **95% CI** | **p value**** |
| **Recipient age** | | 1.023 | [1.019; 1.027] | < 0.0001 |
| **Recipient gender**  Male  Female | | 1  0.900 | [0.815; 0.993] | 0.0357 |
| **BMI, kg/m²,**  18.5-24.9  <18.5  24.9.0-30.0  >30.0 | | 1  1.342  0.975  1.245 | [1.114; 1.616]  [0.876; 1.086]  [1.077; 1.439] | 0.0003 |
| **Dialysis antecedent** | |  |  |  |
| No | | 1 |  |  |
| Yes | | 1.796 | [1.524; 2.117] | < 0.0001 |
| **Causal Nephropathy** |  | |  |  |
| Glomerulopathy  Vascular nephropathy  Chronic interstitial Nephropathy  Congenital  Polycystic  Uropathy  Diabetes  Other | | 1  1.357  1.065  0.951  0.704  1.022  1.311  1.033 | [1.155; 1.595]  [0.858; 1.321]  [0.657; 1.376]  [0.603; 0.823]  [0.846; 1.235]  [1.126; 1.526]  [0.905; 1.179] | < 0.0001 |
| **Overall HLA mismatch A/B/DR** | | 1.046 | [1.009; 1.085] | 0.0156 |
| **Immunization**  No  Yes | | 1  1.223 | [1.056; 1.415] | 0.0070 |
| **Donor type**  Living  Cerebrovascular death  Other cause of death | | 1  1.537  1.835 | [1.195; 1.977]  [1.058; 3.182] | 0.0027 |
| **Transfusion antecedent**  No  Yes | | 1  1.433 | [1.264; 1.623] | < 0.0001 |
| **Post Tx transfusion**  No  Yes | | 1  1.362 | [1.236; 1.501] | < 0.0001 |

Pre- and post-transplant transfusions are separately analyzed. Parameters with a p value < 0.05 were retained in the model with the exception of cold ischemia time (correlated with donor type) and the presence of anti-CMV antibodies (not significant in the full multivariate model). The number of observations read is 12559 and 7126 used. **Cox-proportional-hazard models used to estimate association of the parameters with transplantation success survival. Values of P<0.05 were considered statistically significant and all tests were two-sided. BMI=Body Mass Index, CI=Confidence Interval, CMV=CytoMegaloVirus, HLA=Human Leucocyte Antigen, HR=Hazard Ratio, Tx=Transplantation.

**Table S5 ǀ** Propensity score analysis**.**

| **A** | **Univariate analyses** | | | | | **Multivariate analyse**  **n = 9365**  **(AUC=0.6909)** | | | | |
| --- | --- | --- | --- | --- | --- | --- | --- | --- | --- | --- |
|  | **N patients** | **N Post transfused patients** | **OR** | **95%CI** | **P-value**** | **N patients** | **N Post transfused patients** | **OR** | **95%CI** | **P-value**** |
| **Age*, yr** | 12559 | 3483 | 1.016 | 1.013-1.019 | < 0.0001 | 9365 | 2515 | 1.013 | 1.009-1.017 | < 0.0001 |
| **Gender***  Male  Female | 7762  4797 | 1929  1554 | 1  1.449 | 1.338-1.569 | < 0.0001 | 5821  3544 | 1413  1102 | 1.446 | 1.306-1.601 | < 0.0001 |
| **BMI, kg/m²**  18.5-24.9  <18.5  25.0-29.9  >30.0  *N missing* | 5549  638  2829  997  *2546* | 1608  210  843  285 | 1  1.203  1.040  0.981 | 1.009-1.433  0.942-1.149  0.845-1.139 | 0.1873 |  |  |  |  |  |
| **Dialysis antecedent** |  |  |  |  |  |  |  |  |  |  |
| No  Yes  *N missing* | 1790  10744  *25* | 477  2997 | 1  1.065 | 0.951 - 1.192 | 0.2754 |  |  |  |  |  |
| **Causal Nephropathy*** |  |  |  |  |  |  |  |  |  |  |
| Glomerulopathy  Vascular  nephropathy  Chronic interstitial  nephropathy  Congenital  Polycystic  Uropathy  Diabetes  Other | 3892  903  587  233  2137  997  1225  2585 | 977  262  168  58  460  253  586  719 | 1  1.220  1.196  0.989  0.819  1.015  2.736  1.150 | 1.038-1.432  0.986-1.451  0.728-1.342  0.722-0.929  0.865-1.191  2.394-3.127  1.027-1.286 | < 0.0001 | 2815  690  442  172  1515  752  992  1987 | 661  189  126  41  312  183  470  533 | 1  1.051  1.124  1.131  0.710  1.112  2.653  1.093 | 0.862-1.282  0.889-1.421  0.777-1.648  0.605-0.834  0.914-1.353  2.263-3.112  0.952-1.256 | < 0.0001 |
| **Anti-CMV**  **antibodies** |  |  |  |  |  |  |  |  |  |  |
| - | 3205 | 812 | 1 |  |  |  |  |  |  |  |
| + | 4947 | 1433 | 1.202 | 1.087-1.329 | 0.0003 |  |  |  |  |  |
| *N missing* | *4407* |  |  |  |  |  |  |  |  |  |
| **Overall HLA mismatch**  A/B/DR  *N missing* | 12537  22 | 3478 | 1.114 | 1.080-1.149 | < 0.0001 | 9365 | 2515 | 1.101 | 1.058-1.145 | < 0.0001 |
| **Immunization**  No  Yes  *N missing* | 8434  948  3177 | 2192  325 | 1  1.486 | 1.288-1.714 | < 0.0001 | 8417  948 | 2190  325 | 1  1.225 | 1.048-1.433 | 0.0109 |
| **Donor type***  Living  Cerebrovascular  death  Other cause of  death | 926  11551  82 | 148  3310  25 | 1  2.111  2.306 | 1.763-2.529  1.396-3.809 | < 0.0001 | 679  8609  77 | 95  2395  25 | 1  1.875  2.687 | 1.482-2.372  1.557-4.637 | < 0.0001 |
| **Cold ischemia time**  <12 h  12-24 h  ≥24 h  *N missing* | 1710  5823  1999  *3027* | 388  1627  572 | 1  1.321  1.366 | 1.164-1.500  1.177-1.585 | < 0.0001 |  |  |  |  |  |
| **Transfusion antecedent***  No  Yes | 10950  1609 | 2597  886 | 1  3.942 | 3.539-4.390 | < 0.0001 | 8176  1189 | 1868  647 | 1  4.104 | 3.604-4.672 | < 0.0001 |

| **B** | **Multivariate Model**  **n = 9365 (2534 events)** | | |
| --- | --- | --- | --- |
|  | **HR** | **95% CI** | **p value**** |
| **Transfusion post-Tx** | 1.363 | [1.264; 1.471] | < 0.0001 |

| **C** | **Patient without transfusion in post Tx**  **(n=2386)** | **Patient**  **with transfusion in post Tx**  **(n=2386)** | **P-value** |
| --- | --- | --- | --- |
| **Age, yr**  Mean (SD) | 50.7 (12.9) | 50.4 (13.4) | 0.3943 |
| Median (IQR) | 52.6 (41.6- 60.2) | 50.4 (40.8-60.5) | 0.6178 |
| **Male sexe, n (%)** | 1341 (56.2%) | 1360 (57.0%) | 0.5789 |
| **Causal Nephropathy** |  |  |  |
| Glomerulopathy  Vascular nephropathy  Chronic interstitial nephropathy  Congenital  Polycystic  Uropathy  Diabetes  Other | 666 (27.9%)  171 (7.2%)  136 (5.7%)  37 (1.6%)  293 (12.3%)  178 (7.5%)  412 (17.3%)  493 (20.7%) | 643 (26.9%)  186 (7.8%)  116 (4.9%)  40 (1.7%)  307 (12.9%)  177 (7.4%)  411 (17.2%)  506 (21.2%) | 0.8621 |
| **Overall HLA mismatch**  **A/B/DR,**  mean ±SD  median (IQR) | 3.5 (1.2)  4.0 (3.0- 4.0) | 3.5 (1.3)  4.0 (3.0-4.0) | 0.3107  0.2144 |
| **Immunization**  No  Yes | 2057 (86.2%)  329 (13.8%) | 2092 (87.7%)  294 (12.3%) | 0.1326 |
| **Donor type, n (%)**  Living  Cerebrovascular death  Other cause of death | 97 (4.1%)  2264 (94.9%)  25 (1.0%) | 95 (4.0%)  2267 (95.0%)  24 (1.0%) | 0.9786 |
| **Transfusion antecedent (%)** | 505 (21.2%) | 525 (22.0%) | 0.4816 |

(A) Analysis of factors associated with post-Tx transfusion. A non-parsimonious multivariate logistic regression model was obtained by entering all parameters with p value <0.05, excepting the parameters identified with a strong correlation (cold ischemia time) and anti-CMV antibodies (not significant in the full multivariate model and lack of many data). (B) Transfusion post-Tx association with transplant failure estimated by the IPTW Cox model. (C) Characteristics of patients according to the administration of a transfusion after Tx after propensity score matching with a caliper of 0.05. *N missing = 0. **Cox-proportional-hazard models used to estimate association of the parameters with transplantation success survival. Values of P<0.05 were considered statistically significant and all tests were two-sided. AUC=Area Under the Curve, BMI=Body Mass Index, CI=Confidence Interval, CMV=CytoMegaloVirus, HLA=Human Leucocyte Antigen, HR=Hazard Ratio, OR=Odds Ratio, Tx=Transplantation.

# **Supplementary Figures**

**Figure S1 ǀ** Correlation matrix between factors associated with transplant failure endpoint in Cox univariate analysis.

|  | Recipient age | Recipient sexe | Recipient BMI | Dialysis history | Initial nephropathy | HLA incompatibility | Anti-CMV Ab | Immunisation | Donor type | Cold ischemia | Transfusion history | Transfusion post-Tx |
| --- | --- | --- | --- | --- | --- | --- | --- | --- | --- | --- | --- | --- |
| Recipient age | 1 | 0,02144 | 0,26535 | 0,06539 | 0,06976 | 0,04922 | 0,14816 | -0.00560 | 0,18405 | 0,14439 | 0,03002 | 0,09206 |
|  |  | 0,0163 | <0.0001 | <0.0001 | <0.0001 | <0.0001 | <0.0001 | 0.6517 | <0.0001 | <0.0001 | 0,0008 | <0.0001 |
|  | 12559 | 12559 | 10013 | 12534 | 12559 | 12537 | 8152 | 6492 | 12559 | 9532 | 12559 | 12559 |
| Recipient sexe | 0,02144 | 1 | 0,06569 | 0,03604 | -0,03673 | 0,04865 | -0,08593 | -0.26271 | -0,0032 | -0,03411 | -0,02522 | -0,08187 |
|  | 0,0163 |  | <0.0001 | <0.0001 | <0.0001 | <0.0001 | <0.0001 | <0.0001 | 0,72 | 0,0009 | 0,0047 | <0.0001 |
|  | 12559 | 12559 | 10013 | 12534 | 12559 | 12537 | 8152 | 6492 | 12559 | 9532 | 12559 | 12559 |
| Recipient BMI | 0,26535 | 0,06569 | 1 | 0,032 | 0,00943 | 0,00632 | 0,03591 | -0.01119 | 0,05919 | 0,06065 | -0,0144 | -0,00822 |
|  | <0.0001 | <0.0001 |  | 0,0014 | 0,3454 | 0,5274 | 0,0051 | 0.4297 | <0.0001 | <0.0001 | 0,1498 | 0,411 |
|  | 10013 | 10013 | 10013 | 9991 | 10013 | 9998 | 6086 | 4980 | 10013 | 7257 | 10013 | 10013 |
| Dialysis history | 0,06539 | 0,03604 | 0,032 | 1 | -0,01895 | -0,00391 | 0,05367 | 0.02840 | 0,13289 | 0,10644 | 0,0596 | 0,00974 |
|  | <0.0001 | <0.0001 | 0,0014 |  | 0,0339 | 0,6621 | <0.0001 | 0.0223 | <0.0001 | <0.0001 | <0.0001 | 0,2753 |
|  | 12534 | 12534 | 9991 | 12534 | 12534 | 12512 | 8133 | 6476 | 12534 | 9509 | 12534 | 12534 |
| Initial nephropathy | 0,06976 | -0,03673 | 0,00943 | -0,01895 | 1 | 0,0029 | -0,06385 | -0.02186 | 0,01467 | 0,02164 | -0,02688 | -0,04855 |
|  | <0.0001 | <0.0001 | 0,3454 | 0,0339 |  | 0,7452 | <0.0001 | 0.0782 | 0,1001 | 0,0346 | 0,0026 | <0.0001 |
|  | 12559 | 12559 | 10013 | 12534 | 12559 | 12537 | 8152 | 6492 | 12559 | 9532 | 12559 | 12559 |
| HLA incompatibility | 0,04922 | 0,04865 | 0,00632 | -0,00391 | 0,0029 | 1 | 0,00728 | -0.10779 | 0,17725 | -0,00201 | -0,00743 | 0,06098 |
|  | <0.0001 | <0.0001 | 0,5274 | 0,6621 | 0,7452 |  | 0,5113 | <0.0001 | <0.0001 | 0,8447 | 0,4055 | <0.0001 |
|  | 12537 | 12537 | 9998 | 12512 | 12537 | 12537 | 8135 | 6479 | 12537 | 9514 | 12537 | 12537 |
| Anti-CMV Ab | 0,14816 | -0,08593 | 0,03591 | 0,05367 | -0,06385 | 0,00728 | 1 | 0.05336 | 0,03919 | 0,01588 | -0,00084 | 0,03971 |
|  | <0.0001 | <0.0001 | 0,0051 | <0.0001 | <0.0001 | 0,5113 |  | <0.0001 | 0,0004 | 0,1549 | 0,9394 | 0,0003 |
|  | 8152 | 8152 | 6086 | 8133 | 8152 | 8135 | 8152 | 5589 | 8152 | 8026 | 8152 | 8152 |
| Immunisation | 0.00034 | -0.22498 | -0.01314 | 0.01753 | -0.01128 | -0.09155 | 0.03965 | 1 | 0.03375 | 0.10472 | 0.09248 | 0.06425 |
|  | 0.9735 | <0.0001 | 0.2663 | 0.0899 | 0.2748 | <0.0001 | 0.0004 |  | 0.0065 | <0.0001 | <0.0001 | <0.0001 |
|  | 9382 | 9382 | 7156 | 9360 | 9382 | 9365 | 5589 | 6492 | 6492 | 6403 | 6492 | 6492 |
| Donor type | 0,18405 | -0,0032 | 0,05919 | 0,13289 | 0,01467 | 0,17725 | 0.03919 | 0.02981 | 1 | 0,4272 | -0,00075 | 0,07178 |
|  | <0.0001 | 0,72 | <0.0001 | <0.0001 | 0,1001 | <0.0001 | 0,0004 | 0.0039 |  | <0.0001 | 0,9327 | <0.0001 |
|  | 12559 | 12559 | 10013 | 12534 | 12559 | 12537 | 8152 | 9382 | 12559 | 9532 | 12559 | 12559 |
| Cold ischemia | 0,14439 | -0,03411 | 0,06065 | 0,10644 | 0,02164 | -0,00201 | 0,01588 | 0.09667 | 0,4272 | 1 | -0,00588 | 0,03997 |
|  | <0.0001 | 0,0009 | <0.0001 | <0.0001 | 0,0346 | 0,8447 | 0,1549 | <0.0001 | <0.0001 |  | 0,5657 | <0.0001 |
|  | 9532 | 9532 | 7257 | 9509 | 9532 | 9514 | 8026 | 9231 | 9532 | 9532 | 9532 | 9532 |
| Transfusion history | 0,03002 | -0,02522 | -0,0144 | 0,0596 | -0,02688 | -0,00743 | -0,00084 | 0.08236 | -0,00075 | -0,00588 | 1 | 0,23403 |
|  | 0,0008 | 0,0047 | 0,1498 | <0.0001 | 0,0026 | 0,4055 | 0,9394 | <0.0001 | 0,9327 | 0,5657 |  | <0.0001 |
|  | 12559 | 12559 | 10013 | 12534 | 12559 | 12537 | 8152 | 9382 | 12559 | 9532 | 12559 | 12559 |
| Transfusion post-Tx | 0,09206 | -0,08187 | -0,00822 | 0,00974 | -0,04855 | 0,06098 | 0,03971 | 0.05641 | 0,07178 | 0,03997 | 0,23403 | 1 |
|  | <0.0001 | <0.0001 | 0,411 | 0,2753 | <0.0001 | <0.0001 | 0,0003 | <0.0001 | <0.0001 | <0.0001 | <0.0001 |  |
|  | 12559 | 12559 | 10013 | 12534 | 12559 | 12537 | 8152 | 9382 | 12559 | 9532 | 12559 | 12559 |

Pearson correlation coefficients:

P-value:

The significant correlations were defined by a correlation coefficient ≥ 0.4 associated with a P-value <0.0001: among “cold ischemia” and “donor type”, the parameter “donor type” is selected. ab=antibodies, BMI= Body Mass Index, CMV=Cytomegalovirus, HLA= Human Leucocyte Antigen, Tx=Transplantation.


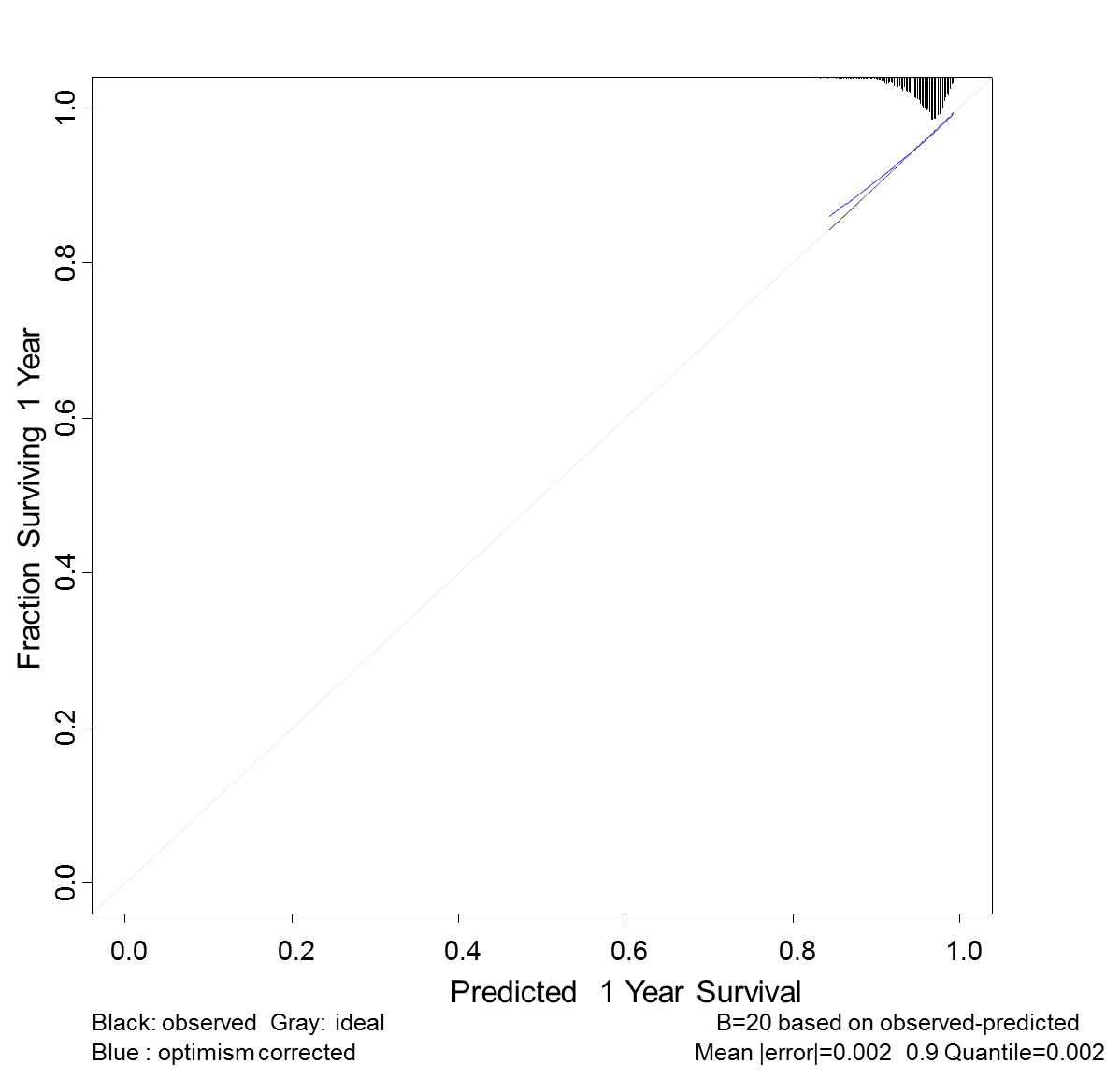
**Figure S2 ǀ** Calibration plots at (A) 1, (B) 3, (C) 5, and (D) 10 years for the final multivariate model.


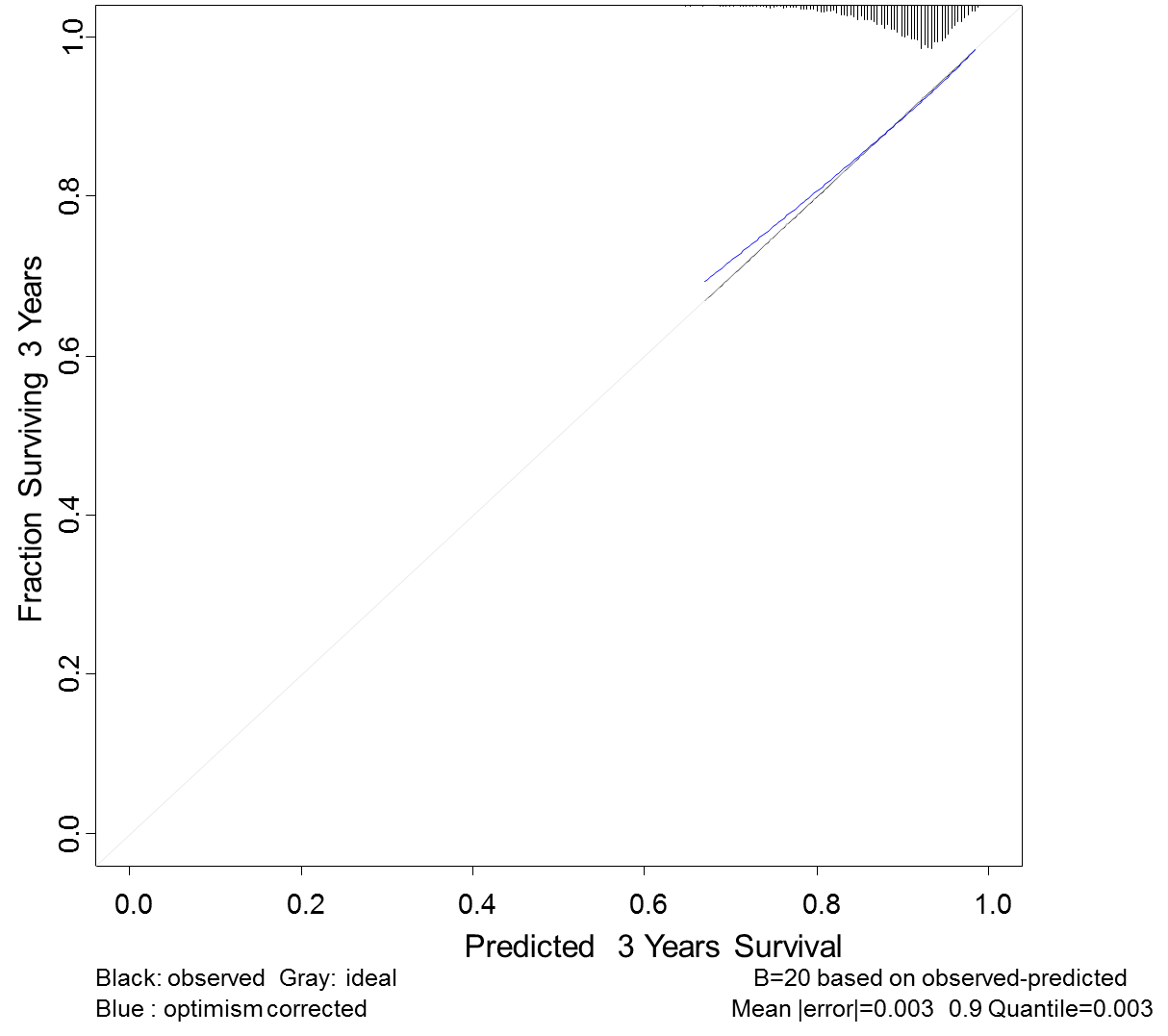


A B


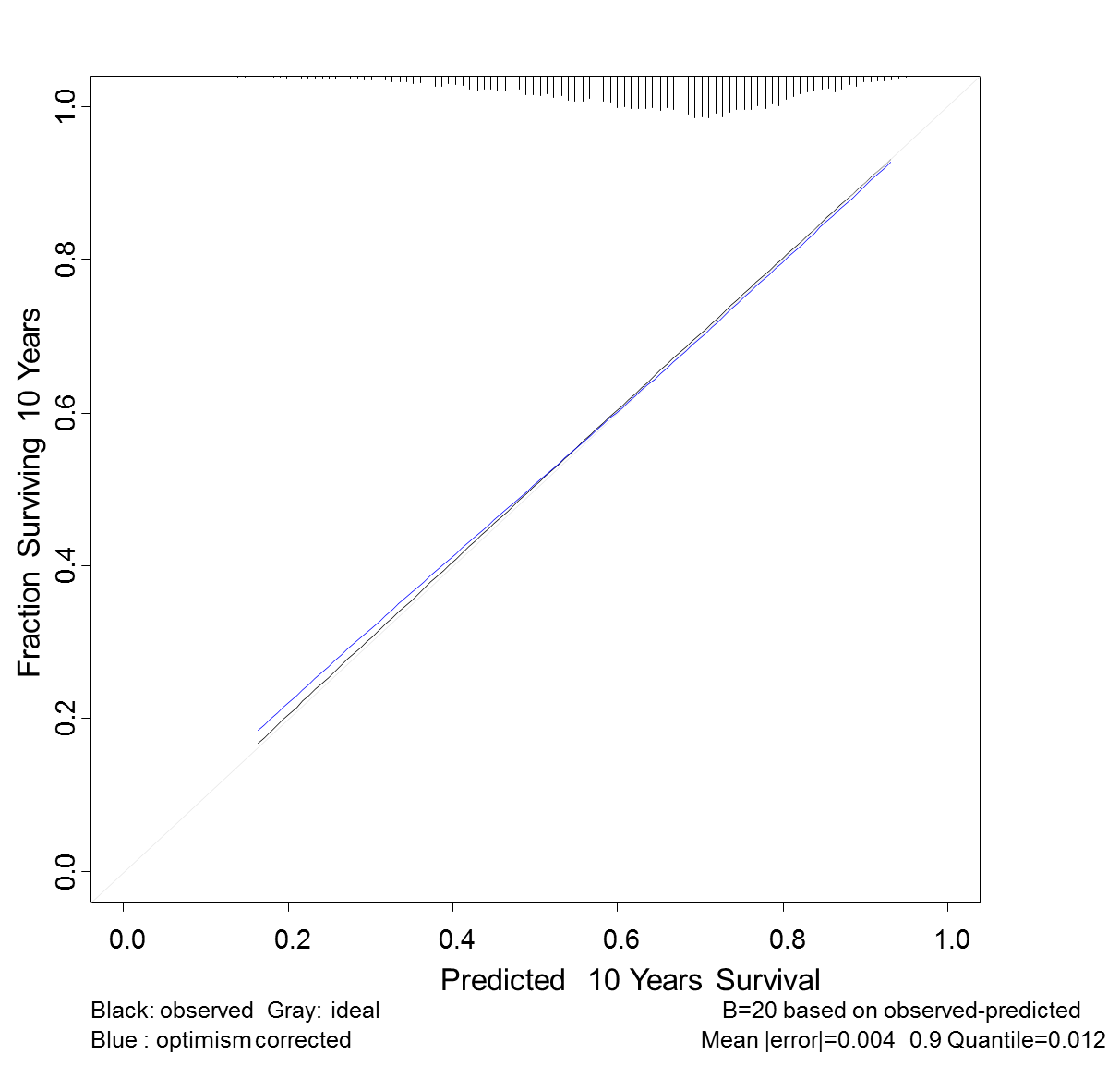

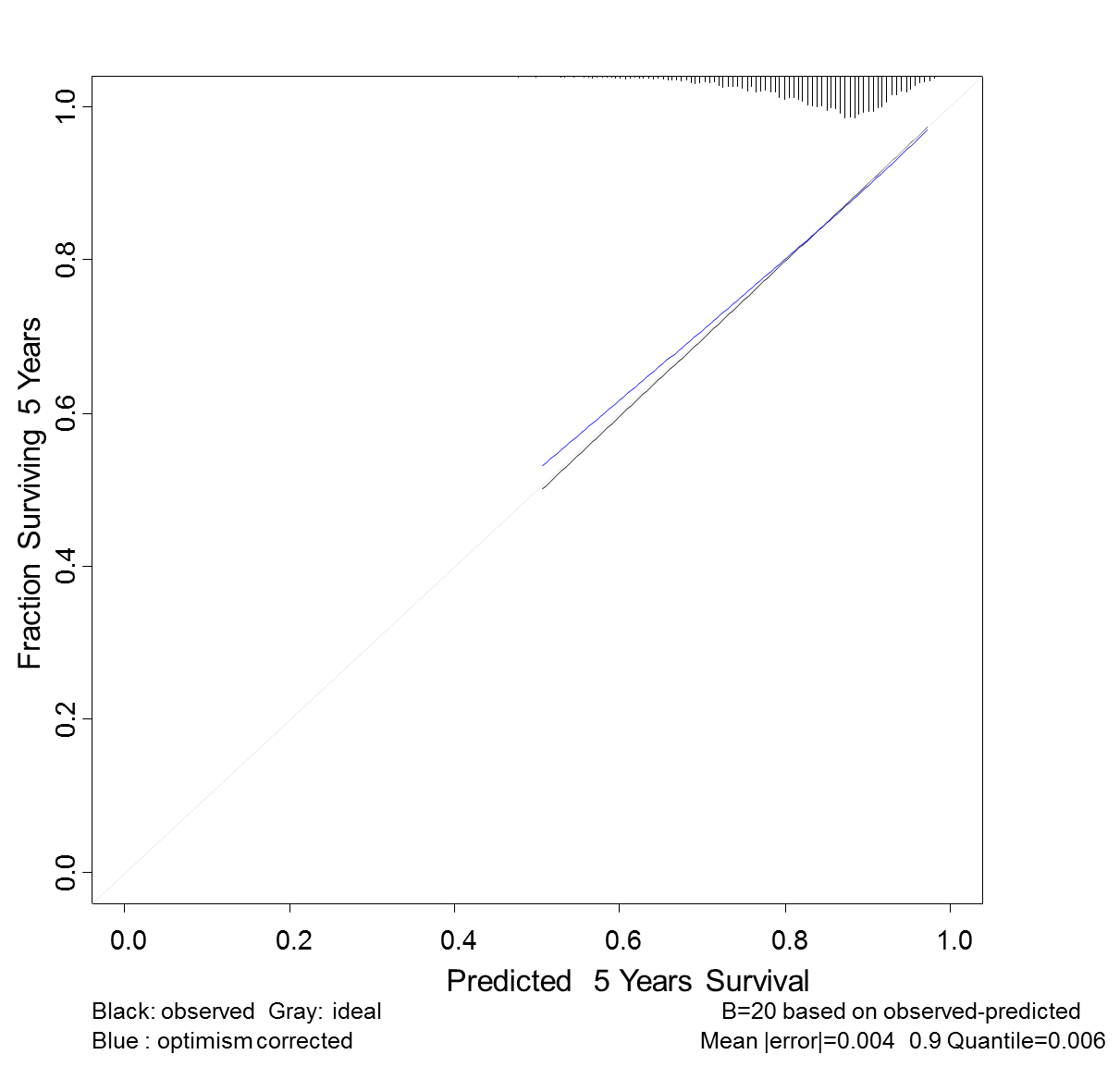


C D

Vertical axis is the observed proportion of patients surviving at time of interest. Black line=observed; Grey line=ideal calibrated model; Blue line=bootstrap corrected estimates (optimism corrected). B=20 repetitions for bootstrap.

**Figure S3 ǀ** Propensity score analysis.


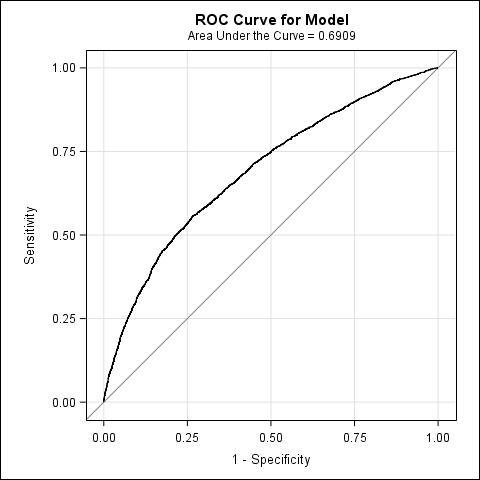
A B


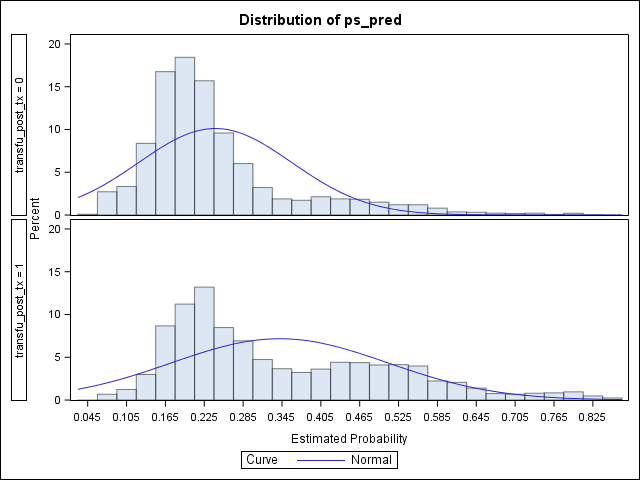


**Distribution of post-Tx transfusion probability**

**Without transfusion**

**With transfusion**

(A) ROC Curve for the model. AUC=0.6909. Black line=observed; Grey line=ideal calibrated model. (B) Distribution of the post-Tx transfusion probability estimated by the final multivariate model in patients with and without post-Tx transfusion. AUC=Area Under Curve, ROC=Receiver Operating Characteristic, Tx=Transplantation.

**Figure S4 ǀ** Kaplan-Meier transplant survival curves for patients according to the post-transplant transfusion after analysis by propensity score and distribution of patients according to a 0.05 caliber.


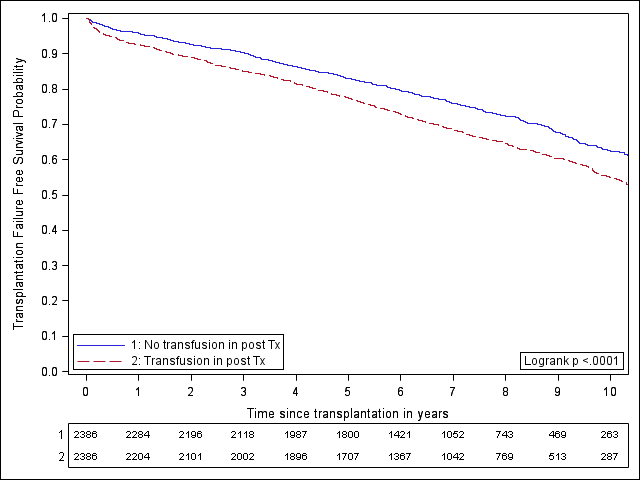


Patients transfused (in red) or not (in blue) are followed for 10 years. TXFFS=Transplantation Failure Free Survival, Tx=Transplantation.
